# A systematic review of the association between ultrasound-detected features and laboratory inflammatory biomarkers in hand osteoarthritis

**DOI:** 10.1080/07853890.2025.2549523

**Published:** 2025-08-20

**Authors:** Omar Alshalawi, Jonathan Fulford, Hussein Al-shaari, Merlisa C. Kemp, Abasiama Dick Obotiba

**Affiliations:** ^a^ Department of Health and Care Professions, Faculty of Health and Life Sciences, University of Exeter, Exeter, UK; ^b^ College of Applied Medical Sciences, Department of Radiological Sciences, Najran University, Najran,KSA

**Keywords:** Osteoarthritis, inflammation, hand joints, ultrasound, biomarkers, erosive, non-erosive

## Abstract

**Objectives:**

To systematically review observational studies for the relationship between ultrasound (US)-detected features and laboratory inflammatory biomarkers in hand osteoarthritis (OA).

**Methods:**

A systematic literature search was performed in MEDLINE, EMBASE, CINAHL, and Web of Science from their inception to June 2025 to identify relevant observational studies. Study quality was evaluated using the Newcastle–Ottawa Scale (NOS), with two independent reviewers validating the papers. Correlation coefficients and corresponding confidence intervals and P values between US-detected features and biomarkers were extracted and analysed.

**Results:**

Out of 5,128 citations, four studies (546 participants, 91.75% female, mean age 56.1–66.3 years) scored >5 on the NOS. Significant correlations (*r* = 0.3–0.57) were found between serum inflammatory markers (e.g. TNF, MIP-β, PDGF-bb, IP-10) and grey-scale synovitis (GSS) specifically in erosive hand OA. No significant correlations were observed between other US-detected features (e.g. power Doppler (PD) signals, osteophytes (OST), effusion, cartilage thickness) and inflammatory biomarkers, with coefficients generally <0.2.

**Conclusion:**

These findings highlight a critical gap in research linking US-detected features and serum inflammatory markers in hand OA. While some evidence suggests that US-detected GSS may reflect subclinical inflammation, particularly in erosive hand OA, inconsistent results across studies underscore the need for larger, standardised research to support phenotyping and inform targeted diagnostic and therapeutic strategies.

## Introduction

Hand osteoarthritis (OA) is a heterogeneous disease that often causes hand pain, deformity, functional disability, and reduced quality of life [1,2]. It can affect multiple joints within the hand, often displaying distinct patterns that tend to occur more frequently in certain population [3]. A particular form, referred to as ‘inflammatory’ or ‘erosive’ hand OA, primarily affects the interphalangeal joints and is marked by abrupt symptom onset, prominent inflammatory features, and radiographic evidence of subchondral erosions, cortical breakdown, and eventual bony ankylosis [4].

Up to date there are no specific diagnostic tests or treatments available for the early molecular or subclinical stages of hand OA [5]. However, similar to OA in other sites, treatment of hand OA is targeted at controlling inflammation and reducing pain, though the role of inflammation in OA remains unclear [6]. It has been indicated that the inability to identify and select patient subgroups who would benefit most from treatments may explain why trials targeted at reducing inflammation have not demonstrated significant and lasting effects on structural changes and symptoms [7]. However, advancements in imaging techniques and soluble biomarkers may offer the necessary tools for effective patient stratification and more insight into the underlying mechanisms of hand OA and modification of treatment strategies.

Although conventional radiography is commonly used to assess morphological abnormalities in hand OA, such as osteophytes (OST), joint space narrowing (JSN), subchondral sclerosis, and bone erosion [8], it has notable limitations. These include its inability to visualize non-bony structures, such as the synovium, ligaments, and tendons, and to provide multi-dimensional assessments of joints [9]. Furthermore, studies have shown that radiographic severity in hand OA does not always correlate with symptoms or clinical signs [10–13]. Ultrasound (US) is a widely accessible, non-invasive imaging technique that allows for real-time visualization of soft tissue structures, such as synovitis, which is an imaging marker of inflammation in OA [7]. It provides a practical advantage over MRI due to its lower cost, faster procedure time, and greater availability in clinical settings [14]. Evidence shows that the US-detected features are associated with signs and symptoms of hand OA [15–18]. Notably, inflammatory changes are reported more frequently in erosive OA than in non-erosive forms, even in joints without erosions, suggesting a systemic inflammatory component [19–22]. Moreover, synovitis detected by power Doppler (PD) signal has been linked to the progression of structural damage, including the formation of new bone erosions, further highlighting the modality’s clinical relevance [23].

Other methods for assessing disease activity in hand OA, such as biomarkers could offer quantitative measures for characterising and monitoring OA in its early stages and may predict its development and progression [24]. Reports show that soluble biomarkers often reflect disease activity more accurately than the current disease status (e.g. severity of radiographic OA) as they are typically produced during the pathophysiological process [25,26]. Several biomarkers such as cartilage oligomeric matrix protein (COMP), C-terminal cross-linking telopeptide of type II collagen (CTX-II), N-terminal propeptide of type II collagen (PIIANP) have been identified as indicators of hand OA [27–31]. Moreover, elevated levels of inflammatory biomarkers, such as C-reactive protein (CRP) and soluble interleukin-2 (IL-2) receptor, have been observed in individuals with erosive hand OA compared to non-erosive hand OA form, supporting the notion that this phenotype reflects a more actively inflammatory disease state [32,33].

Although soluble biomarkers offer quantifiable and precise information on systemic inflammation, they do not provide spatial or joint-specific insights into inflammatory activity. US, despite being operator-dependent, allows for the real-time detection of local synovial inflammation in individual joints. This capability is particularly valuable in a heterogeneous condition like hand OA, where inflammation may be limited to specific joints, especially in erosive phenotypes [23]. Therefore, integrating US-detected features with serum biomarkers may offer a complementary and clinically informative approach to characterising both local and systemic inflammatory processes in hand OA. However, the extent to which US-detected features reflect or correspond with systematic inflammatory biomarkers remains unclear. A number of observational studies have examined these associations US-detected features and reported varying findings [34,35]. To date, no systematic review has synthesised this evidence. Hence, the purpose of this study was to systematically review observational studies investigating the association between US-detected features and laboratory inflammatory biomarkers in hand OA.

## Methods

### Literature search

One reviewer (OS) conducted a systematic literature search in MEDLINE, EMBASE, CINAHL, and Web of Science databases from their inception until June 2025. The search strategy was designed to identify observational studies that investigated the relationship between US-detected features of hand OA and laboratory biomarkers of disease activity in hand OA. The search strategy was structured around key domains related to hand OA, including anatomical terms, condition and clinical signs, US features, and laboratory biomarkers. Keywords such as ‘hand’, ‘interphalangeal’, ‘osteoarthritis’, ‘ultrasound’, ‘biomarker’ and related terms, synonyms, and abbreviations were used. The search protocol was registered in PROSPERO (CRD42023429347).

### Inclusion and exclusion criteria

Studies were included if they investigated the association between US-detected features and systematic markers of inflammation in people with hand OA. Exclusion criteria comprised studies focusing on OA in joints other than the hand, other forms of arthritis such as rheumatoid or psoriatic arthritis, and nonhuman studies. Conference abstracts were excluded due to insufficient data for a systematic review and no language restrictions were applied in the search.

### Data extraction

One investigator (OS) extracted data from the included studies using a predesigned data extraction form. A second investigator (ADO) verified the accuracy of the extracted data. The information collected included the author’s surname, publication year, study setting, study population, sample size, mean age, percentage of female participants, mean body mass index (BMI), diagnostic criteria for hand OA, hand OA phenotype, radiographic and US scoring methods, participant selection criteria, US-detected features examined (e.g. GSS, PD, OST, effusion and cartilage thickness), biomarker sample type, biomarkers investigated (e.g. Clusterin (CLU), CRP, Erythrocyte Sedimentation Rate (ESR), Endothelin-1 (ET-1), -γ Interferon-gamma (IFN), Interleukin-1 beta (IL-1β), Interleukin-1 Receptor Antagonist (IL-1RA), Interleukin-4 (IL-4), Interleukin-8 IL-8 (),Interleukin-9 (IL-9), Interleukin-17 (IL-17), Interferon gamma-induced protein 10 (IP-10), Monocyte Chemoattractant Protein-1 (MCP-1), Macrophage Inflammatory Protein 1-alpha (MIP-1α), Macrophage Inflammatory Protein 1-beta (MIP-β), Platelet-Derived Growth Factor-BB (PDGF-bb), Tumour Necrosis Factor (TNF)) and measures of the association between US-detected features and laboratory biomarkers (e.g. correlation coefficient (r) and odds ratio (OR)).

To evaluate the methodological rigour of US procedures in the included studies, a comparative assessment was undertaken focusing on the number of US time points, the number of raters, the use of blinding, and reported reliability outcomes.

### Quality assessment

The quality of the studies was assessed using the Newcastle Ottawa Scales (NOS) for case-control and cohort studies [36], and a modified NOS for cross-sectional studies [37]. The NOS uses a star rating system, with scores ranging from 0 to 9 stars for case-control and cohort studies, and from 0 to 10 stars for cross-sectional studies. The NOS assesses studies across three domains, including selection of study groups (up to 4 stars for case-control and cohort studies and up to 5 stars for cross-sectional studies), comparability of groups (up to 2 stars), and outcome assessment (up to 3 stars).

### Validation methods

One reviewer (OS) conducted the screening of titles, abstracts, and full texts, as well as data extraction and risk of bias assessment. To validate these assessments, two additional reviewers (JF and HS), who are experts in systematic review methods, independently repeated the evaluations on a randomly selected sample. HS reviewed 10% of the titles (*n* = 414) and abstracts (*n* = 12), while JF reviewed the full texts, assessed the risk of bias, and extracted data from all eligible articles (100%, *n* = 4). Any discrepancies between the reviewers were resolved through discussion and consensus with a third reviewer (ADO).

## Results

### Identification of included studies

**Figure 1.**
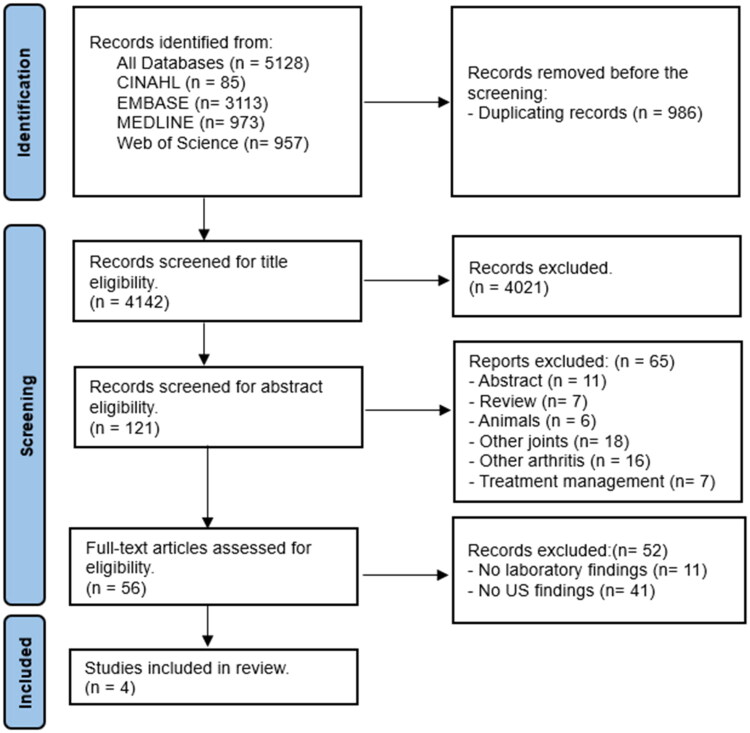
Flow diagram summarising literature search and screening.

The search of the four databases identified 5128 citations. After removing duplicates, and screening of titles and abstracts, 56 articles were left for full-text screening, of which 4 were identified for data extraction and quality assessment. The literature search and screening flowchart is presented in Figure 1.

### Study characteristics

Four cross-sectional studies met the inclusion criteria for this review, which were published between 2018 and 2021. These consisted of a total of 546 participants with 363 confirmed diagnoses of hand OA. Participants were recruited from the rheumatology outpatient clinics and diagnosis was based on the ACR criteria across the four studies. Two studies used consecutive sampling, one used convenience sampling, and one did not specify the sampling method. The mean age ± standard deviation of participants ranged from 56.11 ± 5.36 to 66.3 ± 8.3 years, with the proportion of female participants ranging from 87% to 100%, and BMI ranging from 21 to 37 kg/m^2^. In all included studies, inflammatory biomarkers were investigated from blood serum. A summary of the participant selection criteria, radiographic scoring methods, US-detected features investigated, and laboratory biomarkers investigated is provided in Table 1.

**Table 1.** Study characteristics for included papers.

Author	Sample size	Mean age (Years)	% of female	Mean BMI (kg/m²)	Diagnostic criteria	Radiographic scoring methods	Examined joints	Criteria for participant selection	Ultrasound changes	Laboratory Biomarkers	Statistical test for measures of effect
Atar et al. [38]	58	56.11 ± 5.36	100	31.67 ± 5.33	ACR	K-L	2^nd^ MCPJ (Bilateral)	Exclusion of inflammatory arthritis, infectious arthritis, post-traumatic arthritis, crystal arthritis, avascular necrosis of knee, Charcot disease, carpal tunnel disease, history of intra-articular injection in the knee and hand joints within previous 4 weeks and drug history which could affect the serum ET-1 levels	Cartilage thickness	ET-1	Pearson + Spearman’s correlation
Baloun et al. [34]	104	66.3 ± 8.2	87	27.0 ± 3.5	ACR	Kallman + K-L	DIPJs, PIPJs, MCPJs and 1^st^ CMCJ (Bilateral)	The exclusion criteria for both hand OA patients and controls were the presence of systemic inflammatory disease or cancer.	PD + GSS	Eotaxin IFN-γ IL-1β IL-1RA IL-4 IL-8 IL-9 IL-17 IP-10 MCP-1 MIP-1α MIP-1β RANTES TNF PDGF-bb	Kendall’s tau correlation
Kim et al. [35]	66	57.5 (52 − 63.3)	91	23.4 (21.1 − 25.4)	ACR	K-L	DIPJs, PIPJs and 1^st^ CMCJ (Bilateral)	Excluding subjects with other inflammatory or autoimmune rheumatic diseases	GSS + OST + Effusion	ESR + CRP	Spearman’s correlation
Kropáčková et al. [39]	135	66.3 ± 8.3	89	27.2 ± 4.2	ACR	K-L	DIPJs, PIPJs, MCPJs and 1^st^ CMCJ (Bilateral)	Exclusion of systemic inflammatory disease or cancer.	GSS + PD + OST	CLU	Pearson + Spearman’s correlation

ACR: American College of Rheumatology; CMC: Carpometacarpal joint; CLU: Clusterin; CRP: C-Reactive Protein; DIP: Distal interphalangeal; ESR: Erythrocyte Sedimentation Rate; ET-1: Endothelin-1; GSS: Grey-scale synovitis; HOA: Hand osteoarthritis; IFN-γ: Interferon-gamma; IL-1β: Interleukin-1 beta; IL-1RA: Interleukin-1 Receptor Antagonist; IL-4: Interleukin-4; IL-8: Interleukin-8; IL-9: Interleukin-9; IL-17: Interleukin-17; IP-10: Interferon gamma-induced protein 10; K-L: Kellgren & Lawrence; MCP-1: Monocyte Chemoattractant Protein-1; MIP-1α: Macrophage Inflammatory Protein 1-alpha; MIP-β: Macrophage Inflammatory Protein 1-beta; OST: Osteophyte; PD: Power Doppler; PDGF-bb: Platelet-Derived Growth Factor-BB; PIP: Proximal interphalangeal; TNF: Tumour Necrosis Factor.

### Quality assessment

The average quality score, assessed using the modified NOS, was satisfactory, with all studies scoring 5 or higher out of 10. Detailed results of the quality assessment are provided in Table 2.

**Table 2.** Quality assessment with newcastle–ottawa scale (NOS) scores for included papers [37].

Author	Study design	Selection				Comparability	Outcome		Total score
		Representativeness of the sample	Sample size	Non-respondent	Ascertainment of exposure	Based on design and analysis	Assessment of outcome	Statistical test	
Atar et al. [38]	Cross-sectional	★			★★	★		★	5
Baloun et al. [34]	Cross-sectional	★			★★	★★	★★	★	8
Kim et al. [35]	Cross-sectional	★			★★	★		★	5
Kropackova et al. [39]	Cross-sectional	★			★★	★	★★	★	7

The total NOS score for cross-sectional studies ranges from 0 to 10 stars: Selection (0–5 stars), Comparability (0–2 stars), and Outcome (0–3 stars).

### Assessment of US procedures

The methodological characteristics of US procedures varied across the included studies. All studies employed a single US time point, with either one or two raters conducting the assessments. The number of raters ranged from one sonographer or rheumatologist [35,38] to two sonographers [34,39]. Blinding of raters was reported in two studies, both of which involved multiple assessors. Reported reliability outcomes ranged from moderate to very good [34,38,39] to very good to excellent [35], indicating generally acceptable consistency in scoring despite variations in study design and reporting. Across the four studies in this review, GSS, abnormal PD signal, synovial effusion and OST were evaluated based on the criteria defined by Scheel et al. and Wakefield et al. [40,41]. Cartilage thickness was quantitatively assessed using the criteria proposed by Mathiesen et al. [42].

### Association between laboratory biomarkers and US findings

Three out of four studies investigated the association between GSS and serum inflammatory markers, including CLU, CRP, ESR, IFN-γ, IL-1β, IL-1RA, IL-4, IL-8, IL-9, IL-17, IP-10, MCP-1, MIP-1α, MIP-β, PDGF-bb, and TNF [34,35,39]. The correlation coefficients ranged from − 0.36 to 0.57. The association between abnormal PD signal and inflammatory biomarkers was investigated in two of the studies [34,39]. The correlation coefficients ranged from − 0.21 to 0.21.

Three studies provided reports on the association between US-detected effusion, OST, and cartilage thickness and inflammatory biomarkers such as ESR, CRP, CLU, and ET-1 [35,38,39]. The correlation coefficients ranged from 0.008 to 0.2. Summary of details for the four studies is provided in Table 3.

**Table 3.** Correlation coefficients (r) between US-detected features and inflammatory biomarkers with *p*-values below 0.05, are highlighted in bold with an asterisk (*p < 0.05).

Study Author	Hand OA Status	Laboratory Biomarkers	Ultrasound Changes				
			GSS	PD	OST	Effusion	Cartilage thickness
Atar et al. [38]	–	ET-1	–	–	–	–	Right hand (*r* = 0.109),Left hand (*r* = 0.189)
Baloun et al. [34]	Erosive (*n* = 54)	Eotaxin	r = 0.38*	r= −0.03	–	–	–
		IFN-γ	r = 0.21	r= −0.01	–	–	–
		IL-17	r= −0.22	r= −0.21	–	–	–
		IL-1β	r = 0.19	r= −0.09	–	–	–
		IL-1RA	r = 0.35*	r = 0.07	–	–	–
		IL-4	r = 0.12	r= −0.13	–	–	–
		IL-8	r = 0.30*	r= −0.01	–	–	–
		IL-9	r = 0.14	r= −0.11	–	–	–
		IP-10	r = 0.43*	r= −0.01	–	–	–
		MCP-1	r = 0.40*	r = 0.04	–	–	–
		MIP-1α	r = 0.35*	r = 0.05	–	–	–
		MIP-β	r = 0.50*	r = 0.03	–	–	–
		PDGF-bb	r = 0.44*	r = 0.08	–	–	–
		RANTES	r = 0.43*	r = 0.1	–	–	–
		TNF	r = 0.57*	r = 0	–	–	–
	Non-erosive (*n* = 50)	Eotaxin	r= −0.26	r= −0.13	–	–	–
		IFN-γ	r= −0.06	r= −0.12	–	–	–
		IL-17	r= −0.04	r = 0.01	–	–	–
		IL-1β	r= −0.12	r= −0.15	–	–	–
		IL-1RA	r= −0.19	r = 0.06	–	–	–
		IL-4	r= −0.11	r = 0.02	–	–	–
		IL-8	r= −0.07	r= −0.09	–	–	–
		IL-9	r= −0.16	r = 0.13	–	–	–
		IP-10	r= −0.22	r= −0.14	–	–	–
		MCP-1	r= −0.36*	r= −0.17	–	–	–
		MIP-1α	r= −0.28	r= −0.16	–	–	–
		MIP-β	r= −0.19	r= −0.06	–	–	–
		PDGF-bb	r= −0.27	r= −0.06	–	–	–
		RANTES	r= −0.29	r= −0.05	–	–	–
		TNF	r= −0.22	r= −0.16	–	–	–
Kim et al. [35]	–	ESR	r = −0.047	–	r = 0.008	r = −0.106	–
		CRP	r = −0.165	–	r = 0.080	r = −0.117	–
Kropáčková et al. [39]	Erosive (*n* = 81)	CLU	r = 0.042	r = 0.111	r = 0.189	–	–
	Non-erosive (*n* = 54)	CLU	r = 0.130	r = 0.217	r = 0.214	–	–

CLU: Clusterin; CRP: C-Reactive Protein; ESR: Erythrocyte Sedimentation Rate; ET-1: Endothelin-1; GSS: Grey-scale synovitis; IFN-γ: Interferon-gamma; IL-1β: Interleukin-1 beta; IL-1RA: Interleukin-1 Receptor Antagonist; IL-4: Interleukin-4; IL-8: Interleukin-8; IL-9: Interleukin-9; IL-17: Interleukin-17; IP-10: Interferon gamma-induced protein 10; MCP-1: Monocyte Chemoattractant Protein-1; MIP-1α: Macrophage Inflammatory Protein 1-alpha; MIP-β: Macrophage Inflammatory Protein 1-beta; OST: Osteophyte; PD: Power Doppler; PDGF-bb: Platelet-Derived Growth Factor-BB; TNF: Tumour Necrosis Factor.

Among the included studies, only one study [35] reported adjusting for potential confounding factors, such as age, sex and symptoms duration when analysing the associations between US findings and inflammatory biomarkers. Two studies [38,39] did not report any adjustment for confounders. In the remaining study [34], the authors did not adjust for confounding variables due to the use of bivariate correlation methods (Kendall’s tau) as part of an exploratory analytical approach. However, the authors confirmed that Bonferroni correction for multiple comparisons was applied where appropriate, particularly in analyses involving multiple mediators, to reduce the risk of false positives.

### Discussion

To the authors’ knowledge, this is the first systematic review to examine the relationship between US-detected features and laboratory biomarkers in people with hand OA. One study revealed positive correlations between US-detected inflammation depicted as GSS and 10 serum inflammatory mediators including eotaxin, IL-1RA, IL-8, IP-10, MCP-1, MIP-1α, MIP-1β, PDGF-bb, RANTES, and TNF. However, this positive correlation was found in the erosive but not the non-erosive hand OA group [34]. These suggest that the presence of GSS may indicate an active inflammatory process in erosive hand OA and may be associated with systemic inflammation compared to the non-erosive group. Previous studies demonstrated a higher prevalence of GSS in erosive hand OA in comparison to non-erosive hand OA group [19] and its presence dose-dependently predicts incidence are progression radiographic erosions [43,44], which suggest that erosive hand OA may be a distinct molecular endotype. Evidence suggest that synovial inflammation plays a role in the pathophysiology and symptoms of OA by inducing the local production of pro-inflammatory mediators that contribute to structural joint tissue damage [7,44,45]. Several cytokines that correlated with GSS, including TNF, MCP-1, MIP-1β, and IP-10, play key roles in OA pathogenesis by promoting synovial inflammation and joint tissue degradation. For example, TNF stimulates catabolic enzymes such as matrix metalloproteinases [46], while chemokines like MCP-1 and MIP-1β recruit immune cells to the synovium [47–49]. IP-10 enhances T-cell migration and has been linked to chronic joint inflammation [50]. These mechanisms support the observed US findings and reinforce the notion that erosive hand OA may involve a distinct, cytokine-driven inflammatory process.

However, it is noteworthy that all biomarker correlations in the non-erosive hand OA subgroup, were negative. This disparity in correlation direction between erosive and non-erosive hand OA groups highlights the potential variation in underlying inflammatory pathways. For instance, TNF and chemokines like MCP-1 and IP-10, which correlated positively with GSS in erosive hand OA, were negatively or not correlated in non-erosive disease. This also suggests that the pathogenesis of synovitis may be cytokine-specific and phenotype-related.

In the current review it was found in two studies [34,39] that abnormal PD signal was not correlated with any of the specific inflammatory mediators investigated. This could be explained by the low prevalence of abnormal PD signal in hand OA, which ranges from 2.5% to 4.2% as reported in some studies [18,43,51]. It is noteworthy that although abnormal PD signal is definitive of hypervascularity in the synovium that depicts an active synovial inflammation [52–54], its absence may not completely exclude synovial inflammation [55]. One possible explanation is that active inflammation diminishes or becomes less vascularised as joint damage progresses, although longitudinal studies are needed to confirm this hypothesis.

No significant relationship between GSS and nonspecific inflammatory markers such as CRP and ESR levels was found in one study [35]. This is consistent with findings from a randomised control trial (RCT), that reported no correlation between GSS and CRP levels [56] and reports from a conference abstract that found no correlation between GSS and elevated ESR levels [57]. It has been suggested that early local inflammatory changes might not be severe enough to stimulate a systemic inflammatory response in hand OA patients. Conversely, CRP and ESR levels can be influenced by various inflammatory processes in the body and are not solely elevated by OA [58].

Findings from this review showed that US-detected OST, cartilage thickness and effusion were not associated with specific inflammatory mediators (such as ET-1 and CLU) and nonspecific inflammatory markers (such as CRP and ESR levels) [35,38,39]. This is understandable since structural changes do not always correlate with symptoms and inflammatory changes, as reported in several radiographic OA studies [10,59]. Furthermore, previous studies have also found no association between levels of several serum specific inflammatory mediators (e.g. IL-6, TNF-a, and IL-17) and radiographic changes [30,60]. Thus, the lack of correlation between structural and inflammatory changes detected by US, such as GSS, suggests that synovial inflammation may play a role early in the timeline of structural modifications which is supported by the fact that US can detect inflammatory changes associated with structural changes that occur at later stages of OA [43,61].

This systematic review has some limitations that should be acknowledged. obvious limitation is the small number of included studies and significant methodological differences across these studies. Not all studies examined the same US features or biomarkers. These methodological differences prevented a meta-analysis and limited the ability to draw consistent conclusions. Furthermore, because the associations between biomarkers and US features were examined only within hand OA populations, without incorporating control groups into those analyses, the risk of collider bias remains. This may lead to spurious associations, as both biomarkers and US features could be independently influenced by the underlying disease process. Another limitation relates to the gender imbalance across included studies, with female participants comprising ≥ 87% in each. While this aligns with the epidemiology of hand OA, it may limit the generalizability of findings to male patients. Finally, the key findings of this review are largely derived from a single study that conducted many comparisons between serum biomarkers and US features across erosive and non-erosive hand OA subgroups. While the authors employed an exploratory approach, the absence of formal adjustment for multiple comparisons may increase the risk of false-positive findings, and these results should be interpreted with caution.

## Conclusion

The findings of this review highlight emerging but limited evidence supporting the correlation between US-detected synovitis and a panel of inflammatory cytokines and chemokines such as eotaxin, IL-1RA, IL-8, IP-10, MCP-1, MIP-1α, MIP-1β, PDGF-bb, RANTES, and TNF in in individuals with erosive hand OA. Notably, these correlations were not observed in non-erosive hand OA, suggesting a potentially distinct inflammatory profile in erosive hand OA. No correlations were found with PD signal, synovial effusion, cartilage thickness, and OST with two studies reporting no significant correlations between US features and ESR, CRP, or CLU.

These findings point toward a possible role of US-detected synovitis as an imaging surrogate of subclinical inflammation in erosive hand OA, though its clinical utility remains unconfirmed. The variation in biomarker associations and absence of consistent findings across studies underscore the need for larger, standardised, and longitudinal studies that integrate imaging, biomarker profiling, and clinical outcomes. This could inform efforts to phenotype patients more precisely, guide targeted therapeutic approaches and improve monitoring strategies in hand OA.

## Supplementary Material

Search_strategey - IANN-2025-0771.R2.docx

PRISMA_2020_checklist_Annals of Medicine.docx

## Data Availability

Data will be made available upon reasonable request.
