# Supplementary material for: A systematic review of the association between ultrasound-detected features and laboratory inflammatory biomarkers in hand osteoarthritis
Source: Ann Med. 2025 Aug 20;57(1):2549523. doi: 10.1080/07853890.2025.2549523 (PMC12372511; doi:10.1080/07853890.2025.2549523)
Supplement: Search_strategey - IANN-2025-0771.R2.docx [file IANN_A_2549523_SM7196.docx]

**EMBASE VIA OVID**

1. (Hand* or Interphalangeal* or IP* or DIP* or PIP* or Finger* or Thumb* or Carpometacarpal* or Wrist*).ti,ab.

2. (Osteoarthritis or Degenerative arthritis or OA or Nodes or Heberden* or Bouchard* Osteophyte* or Erosion or effusion or inflammat* or hypertrophy*).ti,ab.

3. (Ultraso* or Sonograph* or Synov* or Doppler).ti,ab.

4. (Laboratory markers or Biomarker or Serum or C-reactive protein or CRP or Erythrocyte sedimentation rate or ESR or Interleukin or IL or Cartilage oligomeric matrix protein or COMP or Type II collagen degradation products or CTX-II or Type I collagen degradation products or CTX-I or C1 or 2C or C2C or Hyaluronic acid or HA or Osteocalcin or Osteoprotegerin or OPG or N-terminal propeptide of type II collagen or PIIANP or Leptin or Adiponectin or Resistin or Pentosidine or Monocyte chemotactic protein or MCP or Myeloperoxidase or MPO or vascular cell adhesion molecule or VCAM).ti,ab.

5. 1 and 2 and 3 and 4

6. limit 5 to human

7. limit 6 to "remove preprint records"

8. limit 8 to "remove Medline records"

**MEDLINE VIA OVID**

1. (Hand* or Interphalangeal* or IP* or DIP* or PIP* or Finger* or Thumb* or Carpometacarpal* or Wrist*).ti,ab.

2. (Osteoarthritis or Degenerative arthritis or OA or Nodes or Heberden* or Bouchard* Osteophyte* or Erosion or effusion or inflammat* or hypertrophy*).ti,ab.

3. (Ultraso* or Sonograph* or Synov* or Doppler).ti,ab.

4. (Laboratory markers or Biomarker or Serum or C-reactive protein or CRP or Erythrocyte sedimentation rate or ESR or Interleukin or IL or Cartilage oligomeric matrix protein or COMP or Type II collagen degradation products or CTX-II or Type I collagen degradation products or CTX-I or C1 or 2C or C2C or Hyaluronic acid or HA or Osteocalcin or Osteoprotegerin or OPG or N-terminal propeptide of type II collagen or PIIANP or Leptin or Adiponectin or Resistin or Pentosidine or Monocyte chemotactic protein or MCP or Myeloperoxidase or MPO or vascular cell adhesion molecule or VCAM).ti,ab.

5. 1 and 2 and 3 and 4

6. limit 5 to human

7. limit 6 to "remove preprint records"

**CINAHL VIA EBSCOhost**

( Hand* OR Interphalangeal* OR (IP) OR (DIP) OR (PIP) OR Finger* OR Thumb* OR Carpometacarpal* OR Wrist* ) AND ( Osteoarthritis Or Degenerative arthritis OR (OA) OR Nodes OR Heberden* OR Bouchard* OR Osteophyte* OR Erosion OR Effusion OR Inflammat* OR hypertrophy* ) AND ( Ultraso* OR Sonograph* OR Synov* OR Doppler ) AND ( Laboratory markers OR Biomarker OR Serum OR C-reactive protein OR CRP OR Erythrocyte sedimentation rate OR ESR OR Interleukin OR IL OR Cartilage oligomeric matrix protein OR COMP OR Type II collagen degradation products OR CTX-II OR Type I collagen degradation products OR CTX-I OR C1 OR 2C OR C2C OR Hyaluronic acid OR HA OR Osteocalcin OR Osteoprotegerin OR OPG OR N-terminal propeptide of type II collagen OR PIIANP OR Leptin OR Adiponectin OR Resistin OR Pentosidine OR Monocyte chemotactic protein OR MCP OR Myeloperoxidase OR MPO OR vascular cell adhesion molecule OR VCAM )

**Web of Science**

<https://www.webofscience.com/wos/woscc/summary/2353ba3f-e404-4fc5-a91a-eb21eab301ee-c7c052fe/relevance/1>

(((TI=(Hand* OR Interphalangeal* OR (IP) OR (DIP*) OR (PIP*) OR Finger* OR Thumb* OR Carpometacarpal* OR Wrist*)) AND TI=(Osteoarthritis Or Degenerative arthritis or OA Or Nodes Or Heberden* Or Bouchard* Osteophyte* Or Erosion or effusion or inflammat* or hypertrophy*)) AND TI=(Ultraso* OR Sonograph* OR Synov* OR Doppler )) AND TI=(Laboratory markers or Biomarker OR Serum OR C-reactive protein OR (CRP) OR Erythrocyte sedimentation rate OR (ESR) OR Interleukin OR (IL) OR Cartilage oligomeric matrix protein OR (COMP) OR Type II collagen degradation products OR (CTX-II) OR Type I collagen degradation products OR (CTX-I) OR (C1) OR (2C) OR (C2C) OR Hyaluronic acid OR (HA) OR Osteocalcin OR Osteoprotegerin OR (OPG) OR N-terminal propeptide of type II collagen OR (PIIANP) OR Leptin OR Adiponectin OR Resistin OR Pentosidine OR Monocyte chemotactic protein OR (MCP) OR Myeloperoxidase OR (MPO) OR vascular cell adhesion molecule OR (VCAM))

**AND**

Letter or Meeting Abstract or Review Article (Exclude – Document Types)
